# Roles of MicroRNAs in Glucose and Lipid Metabolism in the Heart

**DOI:** 10.3389/fcvm.2021.716213

**Published:** 2021-07-22

**Authors:** Hengzhi Du, Yanru Zhao, Huaping Li, Dao Wen Wang, Chen Chen

**Affiliations:** Division of Cardiology, Hubei Key Laboratory of Genetics and Molecular Mechanisms of Cardiological Disorders, Tongji Medical College, Tongji Hospital, Huazhong University of Science and Technology, Wuhan, China

**Keywords:** microRNAs, glucose, lipid, heart, metabolism

## Abstract

MicroRNAs (miRNAs) are small non-coding RNAs that participate in heart development and pathological processes mainly by silencing gene expression. Overwhelming evidence has suggested that miRNAs were involved in various cardiovascular pathological processes, including arrhythmias, ischemia-reperfusion injuries, dysregulation of angiogenesis, mitochondrial abnormalities, fibrosis, and maladaptive remodeling. Various miRNAs could regulate myocardial contractility, vascular proliferation, and mitochondrial function. Meanwhile, it was reported that miRNAs could manipulate nutrition metabolism, especially glucose and lipid metabolism, by regulating insulin signaling pathways, energy substrate transport/metabolism. Recently, increasing studies suggested that the abnormal glucose and lipid metabolism were closely associated with a broad spectrum of cardiovascular diseases (CVDs). Therefore, maintaining glucose and lipid metabolism homeostasis in the heart might be beneficial to CVD patients. In this review, we summarized the present knowledge of the functions of miRNAs in regulating cardiac glucose and lipid metabolism, as well as highlighted the miRNA-based therapies targeting cardiac glucose and lipid metabolism.

## Introduction

Under normal physiological conditions, in order to fulfill a continuous demand for ATP, the heart can metabolize a range of substrates *via* mitochondrial oxidative phosphorylation and substrate level phosphorylation, such as fatty acids, glucose, lactate, and amino acids (1). Before feeding into glycolysis or pentose phosphate pathway, glucose in cardiomyocytes is phosphorylated to glucose-6-phosphate (G6P). Activated by acyl CoA synthetase (ACS), cytosolic free fatty acids can form fatty acyl-CoAs, then could enter mitochondria for oxidation or form ceramides, diacylglycerol (DAG) and triacylglycerol (TAG). Although, the adult hearts mainly use fatty acids for ATP production, hearts demonstrate increased reliance on other substrates such as glucose under pathological conditions (2). The glucose and lipid metabolism in the normal and diseased heart have attracted increasing attentions. Under normal circumstances, except that the sources are lactate, ketone bodies, and amino acids, more than 95% of all substrates are derived from fatty acids and glucose to use for ATP generation for maintaining the function of the heart (2). Importantly, the glucose and lipid metabolism could be changed under pathological injury condition in the heart. It is well accepted, e.g., that hypertrophic heart undergoes a reprogramming process in metabolism, characterized by the increased reliance on glucose metabolism and decreased fatty acids oxidation, which is associated with an increase in glycolysis in the hypertrophied heart (3–5). Cardiac ischemia would lead to poor oxygen supply, inadequate washout of metabolic wastes, and increased glycolytic flux, because the amount of oxygen and metabolic substrates that delivered to the myocardium is insufficient to meet the myocardial energy requirements (6). Abnormal glucose metabolism has also been noted in patients with diabetes mellitus and has been associated with cardiac dysfunction (7). Cardiac glucose uptake in diabetic cardiomyopathy is reduced despite hyperglycemia, which could also contribute to the impaired myocardial glucose utilization in diabetes due to decreased protein level of cardiac GLUT-4 (8). MicroRNAs (miRNAs) are small conserved non-coding RNAs which typically inhibits target mRNA translation or promoting target mRNA degradation in physiological or pathological processes (9, 10). Increasing evidence also suggests nuclear or mitochondrial miRNAs could enhance target gene expression through non-canonical mechanisms (11–13). During several phases of cardiac development, many miRNAs have been detected as important regulators to maintain the formation of normal functional heart tissue (14). miR-17-92 cluster, e.g., was suggested as a critical regulator of cardiomyocyte proliferation and might be a therapeutic target for cardiac repairing and heart regeneration (15). Cardiac miRNAs, such as miR-1 (16), miR133a (17), miR-208a/b (18), and miR-499 (19) could enhance regenerative properties and contribute to the reprogramming of mature non-cardiac cells to cardiomyocytes (20). During the progression of various cardiovascular diseases (CVDs), such as hypertrophy, diabetic cardiomyopathy, and myocardial ischemia, many studies also reported that a variety of miRNAs exerted important functions (21–23). Overexpression of miR-297 was found to accelerate the progression of cardiac hypertrophy by increasing the protein expression of ATF4, Xbps1, chaperon Grp78, and calreticulin, the endoplasmic reticulum stress markers (24). Overexpression of miR-200b was reported to prevent diabetes-induced cardiac functional and structural changes by inhibiting endothelial-to-mesenchymal transition (25). Delivery of antisense microribonucleic acid (anti-miR) against miR-21 improved cardiac function, as well as reduced cardiac fibrosis and hypertrophy in a pig model of myocardial ischemia/reperfusion injury (26).

Importantly, it has been reported that miRNAs possess crucial roles in regulating the glucose and lipid metabolism in a variety of organs. miR-146a, e.g., has been found to improve lipid accumulation as well as glucose and insulin tolerance *via* promoting the oxidative metabolism of fatty acids in the liver (27). In the kidney, through blocking the TLR4/NF-κB pathway, miR-140-5p protected renal tubular epithelial cells against high glucose-induced injury (28). Furthermore, in our recent study, we found miR-320a significantly aggravated diet-induced hyperlipidemia and hepatic steatosis (29). Importantly, the roles of miRNAs in the pathological and physiological regulation of glucose and lipid metabolism in the heart have also been gradually discovered. We previously found that nuclear miR-320a caused lipotoxicity in the diabetic heart and induced cardiac dysfunction by activating transcription of fatty acid metabolic genes (13).

In this review, we focused on the current knowledge to briefly summarize and discuss the regulation of miRNAs in glucose and lipid metabolism during the pathological processes of the heart, and highlighting the potential therapeutic strategies for diseases associated with abnormal cardiac glucose and lipid metabolism.

## Roles of miRNAs in Glucose Metabolism in the Heart

In the heart, miRNAs are critical, which participate in cardiac developmental and pathological processes (30). Blocking the expression of all miRNAs in the cardiovascular system has been reported to lead to death in early pregnancy due to severe heart and vascular development defects (31). To support both electrical and mechanical activities, the heart needs a continuous energy supply which are mainly produced by mitochondrial oxidative phosphorylation under normal circumstances (31). A growing number of studies have also shown that miRNAs played crucial roles in the diseased heart by regulating glucose metabolism.

### Roles of miRNAs in Glucose Transport in Heart

Glucose has been proven to be transported into cardiomyocytes by the glucose transporters, glucose transporter 4 (GLUT-4) or GLUT-1, in the sarcolemma (32). In response to various stresses, such as insulin stimulation, increased energy demand, or ischemia, GLUT-4 and GLUT-1 are transported from intracellular vesicles to the sarcomembrane to increase the rate of glucose uptake and glucose transport (33, 34). Moreover, previous study has shown that the expression of GLUT4 in cardiomyocytes could be regulated by miRNAs. Lu et al. found that overexpression of miR-223 increased glucose uptake *via* increasing the GLUT4 protein expression (35). It is worth mentioning that whether miR-223 regulate glucose uptake in cardiomyocytes only by targeting GLUT4 is not clear and remains to be further studied.

Cardiomyocyte hypertrophy, which is characterized by increased size of cardiomyocytes, is one of the compensatory mechanisms of various CVDs (36). Changes in cardiac energy metabolism and substrate utilization are hallmarks of a hypertrophied heart, including increased dependence on glucose, reduction in fatty acid oxidation rate, and decreased high-energy-phosphate content (37). The shift of substrate preference from fatty acid to glucose is therefore considered beneficial in the hypertrophied heart because glucose has a higher oxygen efficiency for ATP production (38). Moreover, many studies suggested that promoting glucose utilization in the hypertrophied heart could be beneficial (39, 40). Studies were performed to explore whether miRNAs affect glucose transport in the hypertrophic cardiomyocytes. Takahiro et al. found that miR-133 decreased the protein level of KLF15 and the level of its downstream target GLUT4, which was involved in metabolic control in the hypertrophic cardiomyocytes (41). Interestingly, Trotta et al. also found the melanocortin 5 receptor agonism reduced the ratio of GLUT1/GLUT4 glucose transporters on the cell membranes and increased the intracellular PI3K activity in the hypertrophic H9c2 cells by decreasing of the levels of miR-133a (42). The important roles of miR-133 in cardiomyocyte glucose transport were confirmed in different models of cardiac hypertrophy. Moreover, Yang et al. found that miR-200a-5p could disturb glucose metabolism by inhibiting selenoprotein n (Seln), selenoprotein t (Selt), selenoprotein 15 (Sep15), and selenoprotein p1 (Sepp1) expression to alter glucose transport, which eventually induce cardiomyocyte hypertrophy (43).

Diabetic cardiomyopathy is a metabolism-related heart disease, which is characterized by clinical heart failure and diastolic relaxation abnormalities in the early stage in the absence of dyslipidemia, hypertension, and coronary artery disease in the advanced stage (43). Due to decreased protein level of cardiac GLUT-4, cardiac glucose uptake is reduced despite hyperglycemia which could also contribute to the impaired myocardial glucose utilization in diabetes (8). Li et al. (44) revealed that the level of let-7 was increased in the myocardium of diabetic rats compared with non-diabetic rats, whereas improved glucose uptake by inhibiting of the let-7 family miRNAs through GLUT4 pathways. Similarly, Ju et al. (45) found miR-150 reduced the glucose utilization by decreasing the translocation and expression of GLUT-4 in the insulin-resistant cardiomyocytes.

**Figure 1:**
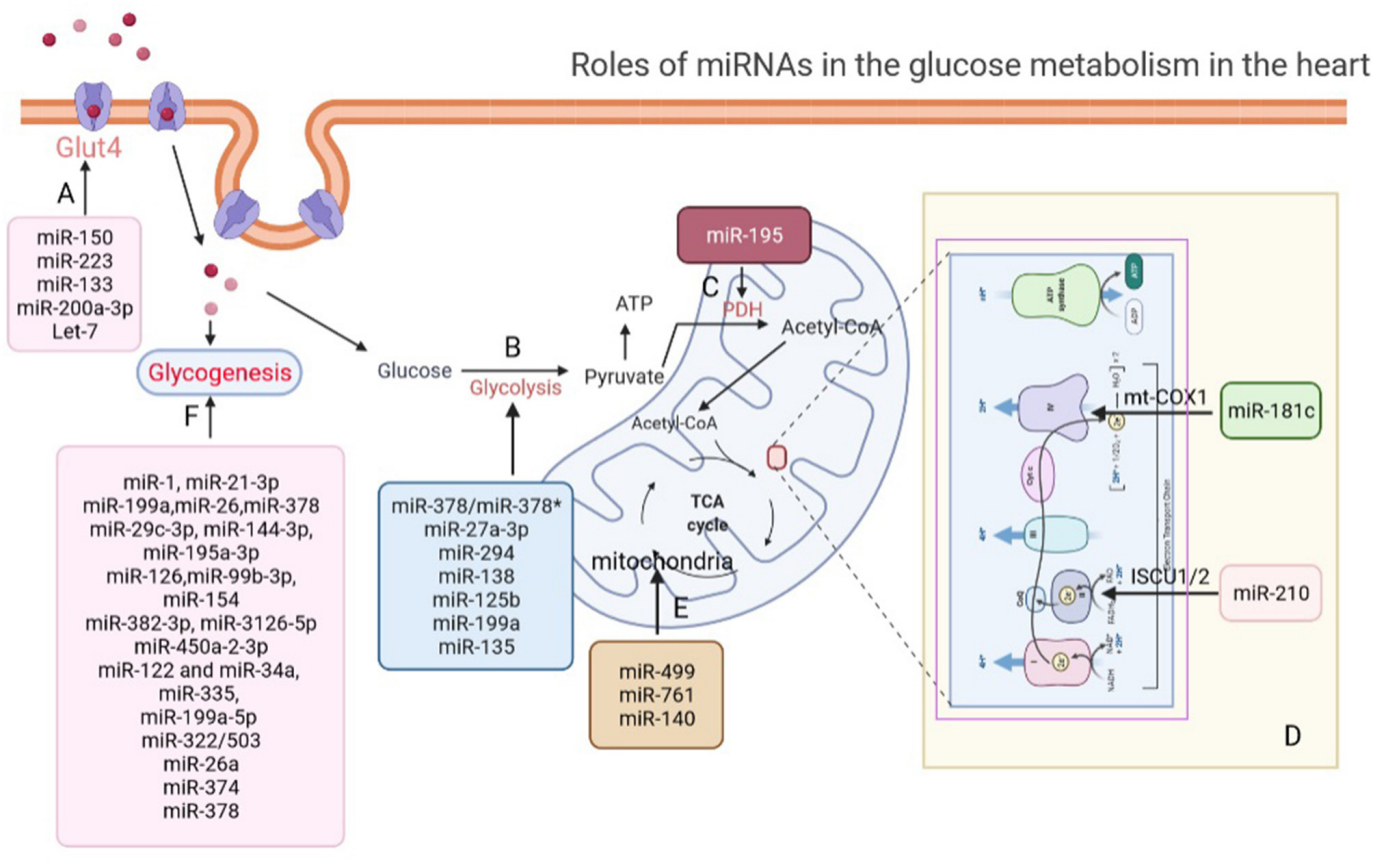
Roles of miRNAs in glucose metabolism in the heart. **(A)** miRNAs regulate glucose transport *via* modulating the expression of GLUT4 in the heart. **(B)** miRNAs participate in glycolysis regulation in the heart. **(C)** miR-195 increased acetylation of PDH to promote pyruvate and NAD^+^ convert into acetyl-CoA. **(D)** miR-181c and miR-210 involve in electron chain complex remodeling in cardiomyocytes by targeting and suppressing mt-COX1 and ISCU1/2. **(E)** miR-499, miR-761, and miR-140 regulate aerobic glucose oxidation by directly affecting mitochondrial function in the heart. **(F)** miRNAs regulate glycogenesis in the heart. GLUT4, glucose transporter type 4; PDH, pyruvate dehydrogenase complex; mt-COX1, cytochrome *c* oxidase subunit 1; ISCU1/2, iron-sulfur cluster assembly proteins.

In conclusion, miRNAs play important roles in the glucose transport in cardiomyocytes under both pathological and physiological processes (Figure 1A).

### Roles of miRNAs in Glycolysis in the Heart

After glucose transport into cardiomyocytes, the first step of glucose catabolism is glycolysis, which produces ATP (46). Although, cardiomyocytes use ATP produced by glucose through the process of glycolysis is limited under normal physiological conditions, glycolysis is thought to facilitate some glucose molecules to be diverted into macromolecular precursors required for lipid, amino acid, and nucleotide biosynthesis and the pentose phosphate pathway (47). Importantly, Mallet et al. (48) suggested miRNAs play important roles in glycolysis of normal cardiomyocytes. They found miR-378 inhibited LDHA expression whereas miR-378^*^ indirectly activated its expression to balance between oxidative phosphorylation and glycolysis in cardiomyocytes. However, the detail mechanism that miR-378 and miR-378^*^ regulate the glycolysis pathway of cardiomyocytes under physiological conditions remains to be further studied.

Most cancer cells rely on aerobic glycolysis, a phenomenon known as the Warburg effect, which differs from the fact that normally differentiated cells rely primarily on mitochondrial oxidative phosphorylation to generate energy for cellular process (47). Similarly, under pathological conditions of the heart, the level of glycolysis would change in contrast to normal physiological state in the heart (49).

During the early stage of myocardial ischemia, glycolysis produces ATP and maintains ionic homeostasis, providing a beneficial effect (50). However, under severe ischemia, glycolysis becomes more harmful than beneficial (51). Importantly, multiple studies have shown that miRNAs played two sides function in glycolysis to regulate cardiac function after myocardial ischemia. On one hand, by performing loss- and gain-of-function experiments and glycolysis stress test, Lei et al. (52) detected that miR-27a-3p restoration enhanced cell viability, depleted cell apoptosis, and promoted glycolysis by targeting TNFR-associated factor 5 (TRAF5) in hypoxia-induced AC16 cells. Borden et al. used AAV delivery system to deliver miR-294 in mice and measured oxygen consumption rates (OCR) and extracellular acidification rates (ECAR). They found that miR-294 could significantly promote proliferation of cardiomyocytes and enhance oxidative phosphorylation and glycolysis that lead to improved cardiac function by targeting Wee1/CyclinB-CDK1 complex after myocardial infarction (53). Bartman et al. (54) performed loss- and gain-of-function experiments and measured ECAR, which revealed that the upregulation of miR-21 facilitated glycolysis and cardioprotection through Per2-dependent mechanisms in myocardial ischemia. Pyruvate dehydrogenase kinase 1 (PDK1), a phosphorylate kinase, phosphorylates pyruvate dehydrogenase leading to elevated anaerobic glycolysis. Zhu et al. (55) observed that miR-138 promoted mitochondrial respiration and inhibited glycolysis through directly targeting PDK1 by measuring lactate product, ECAR, and glycolysis key enzyme, which protected against cardiac cell dysfunction during ischemia. On the other hand, many studies have also shown that miRNAs play a key role in glycolysis to deteriorate cardiac function after myocardial ischemia. Fan et al. showed that miR-125b abolished the beneficial effects of lncRNA-XIST in activating glucose metabolism and cardiomyocyte protection under hypoxia by directly targeting hexokinase 2 (HK2), the key enzyme of glycolysis (56). Similarly, Zhang et al. (57) found that miR-34a inhibited the restoration of glycolysis in dysfunctional cardiomyocytes during ischemia reperfusion (I/R) injury. Moreover, Rane et al. (58) detected that miR-199a was rapidly downregulated in cardiomyocytes and the expression of HK2 and pyruvate kinase-M2 (Pkm2) were enhanced during I/R injury.

It is well-known that there is a prominent metabolic shift from fatty acid oxidation to glucose utilization during cardiac hypertrophy and pathological remodeling, which is associated with an increase in glycolysis in the hypertrophied heart (59, 60). Moreover, it was suggested that the elevation of glycolysis during cardiac hypertrophy and pathological remodeling was through the activation of fructose 2,6-BP and phosphofructokinase-1 (PFK1) in response to cardiac pressure overload (59, 60). It is worth mentioning that miR-135 was found to target PFK1 and inhibit aerobic glycolysis in pancreatic cancer cell, which indicated the possible functions of miRNAs in cardiomyocyte glycolysis (61). However, the role of miRNAs in glycolysis during cardiomyocyte hypertrophy has not been fully revealed (Figure 1B).

### Roles of miRNAs in Aerobic Oxidation of Glucose in the Heart

Glucose can be converted to pyruvate by glycolysis pathway. As the end-product of glycolysis, pyruvate is ultimately transported into mitochondria and is critical for mitochondrial ATP generation. In mitochondria, pyruvate is the main fuel input to drive several major biosynthetic pathways across the citrate cycle and enhance the carbon flux of the citrate cycle (62).

In humans, the mitochondrial pyruvate carrier (MPC), formed by two paralogous subunits, MPC1 and MPC2, is required to deliver pyruvate from the mitochondrial intermembrane space to the mitochondrial matrix (63). In response to cold and heat stress of common carp by performing high-throughput sequencing, Sun et al. found that miRNAs might regulate the expression of MPC in the liver of fish (64). However, the effects of miRNAs on MPC expression and function in the heart remained to be further explored.

After passing through MPC, pyruvate will be oxidized into carbon dioxide by oxidative phosphorylation to ultimately support the generation of ATP (65). Subsequently, pyruvate and NAD^+^ are irreversibly converted into acetyl-CoA, NADH, and carbon *via* the pyruvate dehydrogenase complex (PDH), which serves for bridging glycolytic metabolism in cytoplasm with oxidative phosphorylation and citric acid cycle (66). Importantly, Zhang et al. (67) detected that the expression of miR-195 was increased in failing myocardium, which downregulated the expression of SIRT3 by enhancing global protein acetylation, including PDH complex and ATP synthase directly targeting 3′-untranslated regions that were essential for cardiac energy metabolism (Figure 1C).

In the diseased heart, it was proven that the activity levels of oxidative respiratory chain complex would also change (68). Das et al. indicated that the expression of miR-181c was activated under hypoxic conditions of HF and suppressed cytochrome *c* oxidase subunit 1 (mt-COX1) to involve in electron chain complex IV remodeling in cardiomyocytes, which in turn increased the production of ROS in the heart (69). Similarly, miR-210 suppressed iron-sulfur cluster assembly protein ISCU1/2 expression, which is a chaperone to assemble iron-sulfur clusters and transport these clusters within the functional position in the cell, in hypoxic conditions of heart (70) (Figure 1D).

The tricarboxylic acid (TCA) cycle, a central route for oxidative phosphorylation in cells, depends on the oxidative respiratory chain to fulfill bioenergetic, biosynthetic, and redox balance requirements (71). The oxidative respiratory chain, containing four complexes, establishes an electrochemical gradient over the inner membrane to connect the transport of electrons to oxygen for ATP synthesis (72). In a healthy heart, the various complexes of the oxidative respiratory chain perform their respective functions to maintain the oxidative phosphorylation of glucose supporting cardiomyocytes. Moreover, it has been suggested that many miRNAs play a critical role in regulating mitochondrial function in the heart (Figure 1E). The downregulation of miR-140, as well as the overexpression of miR-499 or miR-761, e.g., could prevent apoptosis and mitochondrial fission in cardiomyocytes *via* regulating mitochondrial fusion/fission-related proteins which led to cardiomyocyte apoptosis, mitochondrial fragmentation, and myocardial infarction (73, 74).

### Roles of miRNAs in Glycogenesis in the Heart

In addition to the glucose consumed by normal metabolism, the excessive glucose can be converted to glycogen for storage through the glycogen synthesis pathway in the heart (75). Cardiac glycogen is an important source of glucose to support high-energy demands of a normal heart (76). Several studies have revealed that miRNAs played an important role in maintaining the balance of glycogen synthesis in the heart. Wei et al. (77), e.g., suggested that deletion of miR-1s led to a large portion in upregulated genes which associated with the cardiac fetal gene programing including glycolysis, cell proliferation, fetal sarcomere-associated genes, and glycogenesis by massively parallel sequencing. Moreover, they found that cardiac-specific overexpression of Errβ, the primary target of miR-1, could induce glycogen storage, cardiac dilation, and sudden cardiac death.

Several key enzymes such as glycogen synthase kinase-3α (GSK3α) and glycogen synthase kinase-3β (GSK3β) are involved in glycogen synthesis (78). It was reported that miRNAs could target GSK3β in some cardiac pathological processes, such as myocardial I/R injury, cardiac hypertrophy, and cardiac fibrosis, which suggested that miRNAs might be involved in glycogen synthesis. Our previous study, e.g., showed that miR-21-3p suppressed HDAC8 expression and decreased phospho-Akt and phospho-Gsk3β expression to attenuate cardiac hypertrophy (79). Moreover, miR-199a (80), miR-26 (81), miR-378 (82), miR-29c-3p, miR-144-3p, miR-195a-3p (83), and miR-126 (84) were reported to target GSK3β in direct or indirect manners during the occurrence and development of pathological cardiac hypertrophy, respectively. Meanwhile, miR-99b-3p (85), miR-154 (86), miR-382-3p, miR-3126-5p, and miR-450a-2-3p (87) were also found to target GSK3β in the pathological process of myocardial fibrosis. miR-122, miR-34a (88), miR-335 (89), miR-199a-5p (90, 91), miR-322/503 (92), miR-26a (93–95), miR-374 (96), and miR-378 (97) were found to target GSK3β in I/R injury model (Figure 1F). However, these studies did not explicitly indicate that these miRNAs were participated in cardiomyocyte glycogen synthesis during these cardiac pathological processes (Table 1).

**Table 1:** Roles of microRNAs in the glucose and lipid metabolism in the heart.

**miRNA(s)**	**Validated targets**	**Key observation**	**References**
miR-223	Glucose transporter type 4 (GLUT4)	Regulate glucose uptake in cardiomyocytes	Cardiovasc Res. 2010;86:410
miR-133	Kruppel-like factor 15 (KLF15)	Reduce the level of the downstream target GLUT4	Biochem Biophys Res Commun.2009;389:315
miR-133a	Glucose transporter type 1/4 (GLUT1/GLUT4)	Increase GLUT1/GLUT4 glucose transporters ratio on the cell membranes	Front Physiol. 2018;9:1475
miR-200a-5p	Stress-related selenoproteins	Lead to glucose metabolism disorder	J Cell Physiol. 2019;234:4095
let-7	Glucose transporter type 4 (GLUT4)	Inhibition of the let-7 family microRNAs improves glucose uptake	Ann Thorac Surg. 2016;102:829
miR-150	Glucose transporter type 4 (GLUT4)	Reduce the glucose utilization	Acta Biochim Biophys Sin. 2020;52:1111
miR-378/miR-378*	Lactate dehydrogenase A (LDHA)	Balance between oxidative phosphorylation and glycolysis in cardiomyocytes	Mol Cell Proteomics. 2014;13:18
miR-27a-3p	TNFR-associated factor 5 (TRAF5)	Promote glycolysis of hypoxia-induced AC16 cells	Life Sci. 2020;262:118511
miR-294	Wee1/CyclinB-CDK1 complex	Enhance oxidative phosphorylation and glycolysis after myocardial infarction	Circ Res. 2019;125:14
miR-21	Period circadian clock 2 (PER2)	Facilitates glycolysis and cardioprotection	PLoS ONE. 2017;12:e0176243
miR-138	Pyruvate dehydrogenase kinase 1 (PDK1)	Inhibit glycolysis but promotes mitochondrial respiration	Biosci Rep. 2017;37
miR-125b	Hexokinase 2 (HK2)	Regulation of lncRNA-XIST in activating glucose metabolism	In vitro Cell Dev Biol Anim. 2020;56:349
miR-34a	Actate dehydrogenase-A (LDHA)	Inhibited the restoration of glycolysis in dysfunctional cardiomyocytes	Biosci Rep. 2017;37
miR-199a	Hexokinase-2 (Hk2); pyruvate kinase-M2 (Pkm2)	Facilitate the upregulation of glycolysis	EMBO J. 2015;34:2671, Circ Res. 2009;104:879
miR-135	Phosphofructokinase-1 (PFK1)	Inhibit aerobic glycolysis in pancreatic cancer cell	Nat Commun. 2019;10:809
miR-195	Pyruvate dehydrogenase complex (PDH)	Increase acetylation of PDH and ATP synthase	Circulation. 2018;137:2052
miR-499, miR-761, miR-140	Mitochondrial fusion/fission proteins	Prevent mitochondrial fission and apoptosis in cardiomyocytes	Free Radic Biol Med. 2013;65:371 PLoS Genet. 2010;6:e1000795
miRNA-181c	Cytochrome *c* oxidase subunit 1 (mt-COX1)	Increase production of ROS in hypoxic conditions of heart	Circ Res. 2012;110:1596
miR-210	Iron-sulfur cluster assembly proteins ISCU1/2	Suppress the iron-sulfur cluster assembly proteins ISCU1/2	Cell Death Dis. 2014;5:e1090
miR-1s	Estrogen-related receptorβ (ERRβ)	Lead to glycogen storage, cardiac dilation, and sudden cardiac death	Cell Res. 2014;24:278
miR-21-3p	Histone deacetylase 8 (HDAC8)	Attenuate cardiac hypertrophy	Cardiovasc Res. 2015;105:340
miR-199a	Glycogen synthase kinase-3β (GSK3β)	Involved in glycogen synthesis	Cell Death Differ. 2017;24:1205
miR-26			J Cardiovasc Pharmacol. 2013;62:312
miR-378			J Biol Chem. 2013;288:11216
miR-29c-3p, miR-144-3p, and miR-195a-3p			J Cell Physiol. 2016;231:1771
miR-126			Cell Mol Life Sci. 2013;70:4631
miR-99b-3p	Glycogen synthase kinase-3β (GSK3β)	Involved in the pathological process of myocardial fibrosis	Acta Pharmacol Sin. 2021;42:715
miR-154			Eur Rev Med Pharmacol Sci. 2018;22:2052
miR-382-3p, miR-3126-5p, and miR-450a-2-3p			J Thorac Dis. 2020;12:5617
miR-122 and miR-34a	Glycogen synthase kinase-3β (GSK3β)	Involved in I/R injury	Biol Trace Elem Res. 2020;196:1
miR-335			J Cell Mol Med. 2019;23:8420
miR-199a-5p			Mol Med Rep. 2019;19:5335-5344Cell Physiol Biochem. 2016;39:1021
miR-322/503			Am J Physiol Cell Physiol. 2019;317:C253
miR-26a			Eur Rev Med Pharmacol Sci. 2020;24:2659 Yonsei Med J. 2018;59:736 Eur Rev Med Pharmacol Sci. 2019;23:7073
miR-374			Cell Physiol Biochem. 2018;46:1455
miR-378			Cardiovasc Res. 2013;100:241
miR-130a, miR-134, miR-141, miR-199a, miR-363, miR-152, and miR-342-3p	Fatty acid translocase (FAT)/CD36	Involved in fatty acids transport	Oncotarget. 2016;7:28806
miR-16, miR-22, miR-26a, and miR-223	Fatty acid translocase (FAT)/CD36	Regulate fatty acid transport	Exp Hematol. 2007;35:551
miR-320	Fatty acid translocase (FAT)/CD36	Increase transportation of fatty acid into diabetic cardiomyocytes	Circ Res. 2019;125:1106
miR-200b-3p	Fatty acid translocase (FAT)/CD36	Regulate fatty acids transport and activate PPAR-γ signaling pathway	J Cell Biochem. 2019;120:5193
miR-197, miR-146b	Fatty acid binding protein (FABP4)/carnitine palmitoyltransferase 1B (CPT1B)	Suppress genes that drive FAO in primary cardiomyocytes	Sci Transl Med. 2018;10
miR-30c	Peroxisome proliferator–activated receptors (PPARs)	Improved lipid and glucose utilization, reduce excessive ROS production	Cardiovasc Diabetol. 2019;18:7
miR-483-3p	Growth/differentiation factor-3 (GDF-3)	Modulated the capacity of adipocytes to store lipids and differentiate	Cell Death Differ. 2012;19:1003
miR-107	Cyclin-dependent kinase 6 (CDK6)	Attenuate differentiation and lipid accumulation	Mol Cell Endocrinol. 2019;479:110
miR-494-3p	Peroxisome proliferator-activated receptor γ (PPARγ)	Prevented TG synthesis, uptake, hydrolysis, and storage in the heart	Eur Heart J. 2019;40:997
miR-451	Calcium-binding protein 39 (Cab39)	Ameliorate palmitate-induced lipotoxicity in cardiomyocytes	Circ Res. 2015;116:279

## Roles of miRNAs in Lipid Metabolism in the Heart

The heart uses ketone bodies, lactate, glucose, fatty acids, and amino acids as energy-providing substrates, among which more than 70% of all substrates are derived from fatty acids to generate ATP in adult heart (98). Moreover, many studies indicate that miRNAs are essential for lipid metabolism in the heart.

### Roles of miRNAs in Fatty Acids Transport in the Heart

Fatty acids (FAs) from albumin or lipoprotein triacylglycerol enter cardiomyocytes through passive diffusion or by protein carrier including fatty acid translocase (FAT)/CD36, fatty acid transport protein (FATP), and plasma membrane isoform of fatty acid-binding protein (FABPpm) (99, 100). Importantly, CD36 could translocate FAs across the membrane of cardiac myocytes. Many studies suggested that 50–60% of FA uptake and oxidation in heart was facilitated by FAT/CD36-mediated transport (101, 102). Unlike FATP or FABPpm, in the regulatory control of FA uptake, CD36 can translocate among the intracellular endosome, the sarcolemmal membrane, and the membrane to promote FA uptake (101, 102).

**Figure 2:**
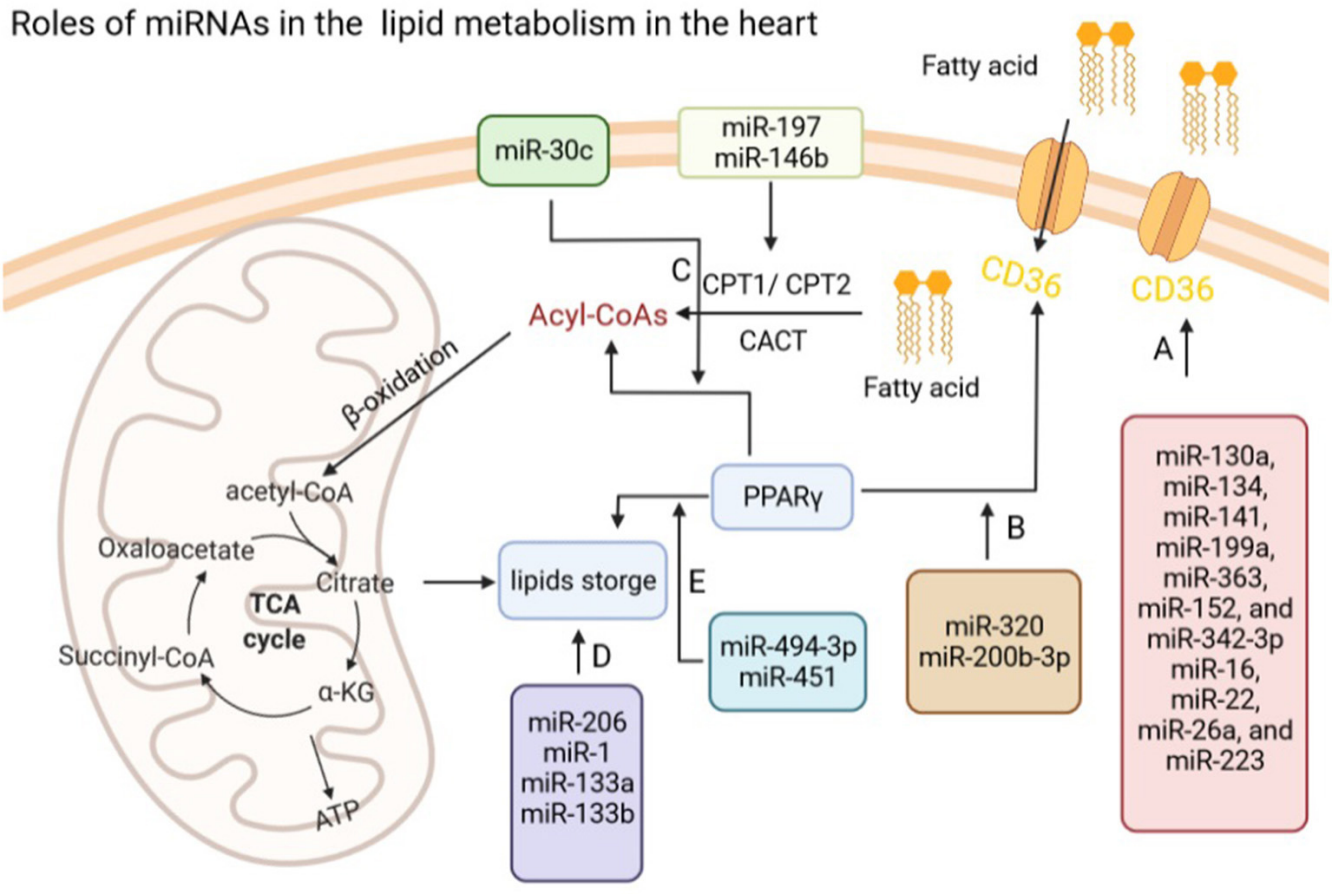
Roles of miRNAs in lipid metabolism in the heart. **(A)** miRNAs regulate fatty acids transport *via* modulating the expression of CD36 in the heart. **(B)** miR-320 and miR-200b-3p regulate fatty acid transport *via* PPARγ. **(C)** miR-197 and miR-146b modulate Acyl-CoAs by CPT1/2, while miR-30c regulates the production of Acyl-CoAs through targeting PPARγ. **(D)** miR-206, miR-1, and miR-133a/b influence lipids storage in the heart. **(E)** miR-494-3p and miR-451 regulate lipids storage *via* modulating the expression of PPARγ in the heart. CD36, fatty acid translocase (FAT)/CD36; CPT1/2, carnitine palmitoyltransferase 1/2; PPARγ, peroxisome proliferator-activated receptors (PPARγ).

Many studies have reported that different miRNAs targeted CD36 mRNA and regulated its expression at the posttranscriptional level in a tissue-specific manner (103, 104). For example, in the process of bone marrow cell differentiation to the monocytic-macrophage line, Zhou et al. (105) reported that CD36 was increased and its expression level was associated with seven miRNAs, including miR-134, miR-130a, miR-199a, miR-141, miR-152, miR-363, and miR-342-3p. During the erythropoiesis, miR-26a, miR-22, miR-16, and miR-223 were detected to correlate with the level and appearance of CD36 as an erythroid surface antigen by performing the expression profiling of miRNAs (106). It should be noticed that the role of miRNAs targeting CD36 to regulate FA transport in the normal heart remains to be further investigated (Figure 2A).

In the cardiac pathological processes, the FA transport would be changed. During diabetic cardiomyopathy, e.g., energy source will be shifted from glucose to FAs and the FA transport is enhanced to meet the increasing demand of ATP (107). However, lipid accumulation in cardiomyocytes might eventually lead to lipid toxicity that promote contractile abnormalities and cell death (108, 109). Therefore, exploring whether miRNAs are involved in FA transport in diabetic hearts might be helpful in discovering potential therapeutic strategies for diabetes-induced cardiac dysfunction (Figure 2B). Our previous research showed that miR-320 could target the CD36 promoter directly resulting in increased transportation of FAs into diabetic cardiomyocytes *via* enhancing CD36 transcription by forming a complex with Ago2 (13). In addition, Xu et al. (110) also found miR-200b-3p expression was significantly reduced in diabetic cardiomyopathy tissues and cells, which could target CD36 directly to reduce cardiomyocytes apoptosis in diabetic cardiomyopathy.

### Roles of miRNAs in Fatty Acid Oxidation in the Heart

FAs are the main energy source in adult heart. Acyl-CoA synthetases (ACS) activate cytoplasmic FAs to acyl-CoA esters and then imported into mitochondrion by two acyltransferases, carnitine acyl-carnitine translocase (CACT) and carnitine palmitoyl transferases 1 and 2 (CPT1 and CPT2). Acyl-CoAs are degraded *via* β-oxidation, finally producing acetyl-CoA to fuel the tricarboxylic acid (TCA) cycle inside the mitochondrion (111). Impaired fatty acid oxidation (FAO) led to the decrease of the capacity for ATP production and accumulation of toxic lipid intermediates in the heart, while enhanced FAO was associated with increased oxidative stress (2, 112).

Ekaterina et al. found that miR-146b and miR-197 were upregulation in the failing right ventricular of pulmonary arterial hypertension patients and suppressed genes that drive FAO (CPT1b and FABP4) in primary cardiomyocytes (113). Peroxisome proliferator-activated receptors (PPARs), a class of ligand-activated nuclear receptors, control FAO enzymes expression, while PPARγ coactivator-1β (PGC-1β) is an important coactivator of PPARs (114–116). Our previous work showed that exogenous miR-30c delivery improved lipid and glucose utilization, reduced excessive ROS production and thereby attenuated cardiac dysfunction *via* PGC-1β/PPARα signals in a mouse model of diabetic cardiomyopathy (117) (Figure 2C).

### Roles of miRNAs in Lipid Storage in the Heart

Cardiomyocytes could reserve multiple energy substrates, among which accumulation of non-polar and polar lipids could activate intracellular signaling pathways (98). The FAs are stored as triacylglycerol (TAG) in lipid droplets. The accumulation of excess lipids is prevented by the physiological balance of lipid uptake and oxidation (98). However, various processes that affect this balance might lead to hypoxia, obesity, diabetes mellitus, sepsis, cardiac dysfunction, and even heart failure.

Many studies suggested that miRNAs play an important role in the lipid storage (Figure 2D). For example, Pegoraro et al. (118) suggested that miR-133a, miR-133b, miR-1, and miR-206, might be useful biomarkers for neutral lipid storage disease with myopathy. Ferland-McCollough et al. (119) demonstrated that miR-483-3p modulated the capacity of adipocytes to store lipids and differentiation by manipulating growth/differentiation factor-3 expression. Moreover, overexpression of miR-107 attenuated differentiation and lipid accumulation in pre- and mature human adipocytes of Simpson-Golabi-Behmel syndrome *via* regulating CDK6 and Notch signaling (120).

Considering the heart, metabolic cardiomyopathy is the main cause of heart failure in obese patients characterized by lipotoxic damage and intramyocardial triglyceride (TG) accumulation (121). JunD could enable transcription of genes involved in TG synthesis, uptake, hydrolysis, and storage by directly binding to PPARγ promoter. Costantino et al. found that miR-494-3p prevented TG synthesis, uptake, hydrolysis, and storage in the heart from diet-induced obese mice by suppressing JunD/PPARγ signaling, which was also associated with myocardial left ventricular (LV) dysfunction and TG accumulation (122). Besides, increased miR-451 were reported in the mouse heart with high-fat diet (HFD), while loss of miR-451 alleviated palmitate-induced lipotoxicity in cardiomyocytes *via* inhibiting calcium-binding protein 39 (Cab39), which is an AMP-activated protein kinase (AMPK) upstream kinase (123) (Figure 2E).

## Potential miRNA-Based Therapy in CVDs

Therapeutic strategies targeting miRNAs for CVDs have been highlighted in many studies (124). For instance, miR-15 family was found to be consistently upregulated during postnatal development of the heart and CVDs, knockdown of the miR-15 family by anti-miRNAs could increase the number of mitotic CMs and reduce the infarct size after ischemia-reperfusion injury in neonatal mice (125–127). As miRNAs can affect different genes simultaneously to alter glucose and lipid metabolism in the pathological processes of diseased heart, they attracted increasing attentions for potential therapeutic targets and treatments (128, 129).

Various strategies were developed for the delivery miRNAs into cardiomyocytes. A novel technique called ultrasound-mediated sonoporation, which carry genetic material to target sites, using albumin-shelled microbubbles, has been considered for miRNA delivery in the myocardium (130). Importantly, Su et al. (131) has used this approach to prevent coronary microembolization-induced cardiac dysfunction by delivering hsa-miR-21-5p in pig myocardium by ultrasound-targeted microbubble. In addition, local injection is a nicely method to overcome the systemic effects on other organs and obtain better transfection efficiency. Many trials have attempted to inject miRNAs *via* intramyocardial or intracoronary directly during heart surgeries (132). Moreover, with the development of new techniques such as positron emission tomography and electromechanical mapping, clinicians can achieve high efficiency around the site of injection by better targeting site of myocardial ischemia (7, 133).

However, there are limitations of miRNA-based therapy, which should be solved before clinical use. Compared with the physiological miRNA expression levels, gain- and loss-of function assays using synthesized oligonucleotides often induce very high abundance of miRNA into the cells, which may lead to irreproducible and misguided interpretation of the results. Most of miRNA studies have been focused on site-specific phenotypic effects *in vivo*, which might ignore the signaling pathways responsible for their effects on other organs and the whole genome targets. Moreover, the off-target effects cannot be ignored. Thus, studies are needed to use both site-specific deliveries and systemic approach to focus on the *in vivo* miRNA effects.

## Conclusion

An increasing number of studies have provided important clues of miRNAs and their potential roles in the glucose and lipid metabolism in CVDs. Current studies revealed the biological and pathological process that miRNAs involved, which might broaden the treatment strategies for CVD patients with or without metabolism disorders. In this review, we systematically described the effects of miRNAs on the glucose and lipid metabolism in cardiomyocytes and compared the advantages and limitations in miRNA-based therapy in CVDs. In addition, we provided a summary table to better illustrate the various miRNAs that participate in glucose and lipid metabolism in the heart. However, considering the multiple targets of one certain miRNA, there are still uncertainties that remain regarding the systemic effects of miRNAs on other organs and biological processes. In summary, miRNAs play critical roles in the regulation of glucose and lipid metabolism in CVDs. MiRNAs and miRNA-based therapies are one of the most promising innovative applications in CVD treatment in the future.

## Author Contributions

All authors listed have made a substantial, direct and intellectual contribution to the work, and approved it for publication.

## Conflict of Interest

The authors declare that the research was conducted in the absence of any commercial or financial relationships that could be construed as a potential conflict of interest.
